# Sesquiterpene lactones and cancer: new insight into antitumor and anti-inflammatory effects of parthenolide-derived Dimethylaminomicheliolide and Micheliolide

**DOI:** 10.3389/fphar.2025.1551115

**Published:** 2025-02-20

**Authors:** Jian Li, Xin Li, Hongwei Liu

**Affiliations:** Department of Thyroid Head and Neck Surgery, Cancer Hospital of Dalian University of Technology, Shenyang, Liaoning, China

**Keywords:** parthenolide, Micheliolide, Dimethylaminomicheliolide, antiinflammatory, anti-cancer

## Abstract

The isolation and application of biological macromolecules (BMMs) have become central in applied science today, with these compounds serving as anticancer, antimicrobial, and anti-inflammatory agents. Parthenolide (PTL), a naturally occurring sesquiterpene lactone derived from Tanacetum parthenium (feverfew), is among the most important of these BMMs. PTL has been extensively studied for its anticancer and anti-inflammatory properties, making it a promising candidate for further research and drug development. This review summarizes the anticancer and anti-inflammatory effects of PTL and its derivatives, with a focus on Micheliolide (MCL) and Dimethylaminomicheliolide (DMAMCL). These compounds, derived from PTL, have been developed to overcome PTL’s instability in acidic and basic conditions and its low solubility. We also explore their potential in targeted and combination therapies, providing a comprehensive overview of their therapeutic mechanisms and highlighting their significance in future cancer treatment strategies.

## 1 Introduction

Medicinal plants have long been revered for their therapeutic properties. These plants produce a wide variety of compounds, both constitutive and secondary metabolites, many of which are biologically active. Aromatic compounds, often phenolic or oxygen-substituted derivatives, are commonly found in plant-based extracts used in traditional medicine (Newman and Cragg, 2020). Sesquiterpene lactones (SLs), a group of plant metabolites, have been utilized for centuries to treat various inflammatory conditions, including high fevers, headaches, stomachaches, toothaches, rheumatoid arthritis (RA), menstrual irregularities, and other inflammatory disorders (Mathema et al., 2012). Parthenolide (PTL), a notable sesquiterpene lactone, is primarily derived from Tanacetum parthenium and exhibits both anti-inflammatory and anticancer properties. The nucleophilic lactone ring, methylene, and epoxide groups of PTL enable rapid interactions with biological targets (Smolinski and Pestka, 2005).

SLs are predominantly sourced from the Asteraceae family and have been recognized since the 1970s for their anti-inflammatory and various other biological activities, including antitumor, cytotoxic, and antibacterial effects (Merfort, 2011). PTL, Micheliolide (MCL), and Dimethylaminomicheliolide (DMAMCL) are key members of the SL family, exhibiting both anti-inflammatory and antitumor activities. However, PTL’s instability in acidic and basic conditions limits its use (Jin et al., 2007). MCL, which is more stable than PTL, exhibits a half-life of 2.64 h, though it remains unstable at room temperature (Zhang et al., 2012). DMAMCL, a prodrug of MCL, continuously releasing MCL over an 8-h period in the plasma. It demonstrates improved stability, stronger activity, and lower toxicity, while also having the ability to cross the blood-brain barrier (Zhang et al., 2012; An et al., 2015; Xi X. N. et al., 2019). The growing body of research on these compounds’ anti-inflammatory and anticancer activities suggests that they hold significant therapeutic potential. This review aims to compile and synthesize available data, providing insights and opening new research avenues.

## 2 PTL: a germacrane sesquiterpene lactone exhibiting potent anti-inflammatory and anticancer properties

### 2.1 Anti-inflammatory mechanisms of PTL

PTL, a potent anti-inflammatory agent, exerts its effects primarily through the inhibition of the Toll-like receptor 4 (TLR4)-mediated activation of Akt, mTOR, and NF-κB pathways. This reduces the production of key inflammatory mediators, positioning PTL as a potential therapeutic for various inflammation-related disorders, including psoriasis (Zhan et al., 2024), RA (Williams et al., 2020), and colon inflammation (Liu Y. J. et al., 2020). PTL has also demonstrated promising results in reducing mortality in patients with severe COVID-19, likely due to its ability to lower IL-6 cytokine levels (Liu Y. J. et al., 2020; Bahrami et al., 2020).

### 2.2 Anticancer mechanisms of PTL

PTL has emerged as the first small molecule identified to target cancer stem cells (Ghantous et al., 2013). Recent studies have revealed PTL’s potential to induce apoptosis in various cancer cell lines, including acute lymphoblastic leukemia (Ortiz-Reyes et al., 2024), melanoma (Dorostgou et al., 2024), lung cancer (Cai et al., 2024), colorectal cancer (Liu Y. C. et al., 2017), and pancreatic cancer (Liu W. et al., 2017; Denda et al., 2024). PTL has shown significant antiproliferative effects in multiple cancer models, including lung cancer, where it suppresses IGF-1R-mediated PI3K/Akt/FoxO3α signaling (Lin et al., 2017) (Table 1). PTL has also been found to inhibit the growth of colorectal cancer cells through the inhibition of USP7/Wnt signaling (Li et al., 2020) and affect cell migration and invasion via the TGF-β1 and NF-κB pathways (Zhu et al., 2019; Kim et al., 2017a). However, the drug’s high lipophilicity and poor solubility in blood plasma limit its oral bioavailability. To overcome these limitations, a water-soluble analogue, Dimethylaminoparthenolide (DMAPT), has been developed, showing improved bioavailability and enhanced therapeutic efficacy (Deraska et al., 2018; Flores-Lopez et al., 2018). Furthermore, DMAPT was also regarded as a potential agent for breast cancer, bladder cancer, and lung cancer (Wang et al., 2017; Shanmugam et al., 2011; Song et al., 2014).

**TABLE 1:** Anti-tumor mechanisms involving inhibition of signaling pathways.

Compound	Target signaling pathway	Effect	Cancer type	Ref
PTL	IGF-1R-mediated PI3K/Akt/FoxO3α	Inhibits lung cancer growth	Lung Cancer	Lin et al. (2017)
PTL	USP7/Wnt	Suppresses colorectal cancer cell growth	Colorectal Cancer	Li et al. (2020)
PTL	TGF-β1 and NF-κB	Inhibits cell migration/invasion and EMT process	Colorectal Cancer	Zhu et al. (2019), Kim et al. (2017a)
MCL	p65/NF-κB	Alkylates p65 cysteine-38, inhibits DNA binding, antagonizes NF-κB	Inflammatory Diseases, Cancer	Zhan et al. (2024)
MCL	PI3K/Akt	Inhibits phosphorylation of Akt, reduces NLRP3 inflammasome activation	Sepsis, Tuberculosis	Yang et al. (2023a), Zhang et al. (2017)
MCL	TrxR	Inhibits thioredoxin reductase, promotes oxidative stress-mediated apoptosis	Hepatocellular Carcinoma	Xu et al. (2022)
MCL	JAK/STAT	Inhibits JAK/STAT signaling pathway, reduces mutant allele burden in Jak2V617F models	Myeloproliferative Neoplasms	Huang et al. (2023)
MCL	EGFR-Akt-Bim	Regulates EGFR-Akt-Bim signaling pathway	Pancreatic Ductal Adenocarcinoma	Yang et al. (2023b)
DMAMCL	Bcl-2	Targets Bcl-2 signaling pathway to induce apoptosis	Glioma Cells	An et al. (2015)
DMAMCL	PAI-1/PI3K/AKT	Suppresses PAI-1/PI3K/AKT pathway, inhibits glioma cell proliferation, invasion, and migration	Glioblastoma	Zanders et al. (2019), Wang et al. (2020)
DMAMCL	STAT3	Inhibits STAT3 phosphorylation, decreases PD-L1 expression	Glioblastoma	Wang et al. (2020)
DMAMCL	STAT3	Inhibits STAT3 phosphorylation, decreases PD-L1 expression	Glioblastoma	Wang et al. (2020)
DMAMCL	PKM2	Promotes tetramerization of PKM2, rewires aerobic glycolysis, suppresses GBM cell proliferation	Glioblastoma	Guo et al. (2019)

### 2.3 Challenges and development of PTL derivatives

Despite the promising antitumor effects of PTL and DMAPT, their lack of selectivity between normal and cancerous cells remains a significant challenge. To address this issue, tumor-targeted formulations such as nanocarriers and liposomes have been developed, which improve drug efficacy while minimizing side effects. For example, the combination of PTL and ginsenoside CK in liposomes has shown improved efficacy in lung cancer treatment (Jin et al., 2018). Furthermore, PLGA-antiCD44-PTL nanoparticles have enhanced drug delivery and bioavailability by selectively targeting leukemic cells (Darwish et al., 2019). However, previous studies have highlighted several disadvantages. For instance, issues such as poor bioavailability and low water solubility have been noted in some studies (An et al., 2022). Another disadvantage is that these biological extracts are obtained from the plants. However, the seasonal availability of plants makes it challenging to consistently source the proper and unique plants needed to obtain these biologically active molecules, posing a significant hurdle in the development of PTL derivatives.

## 3 MCL, synthesized from PTL, exert its anti-inflammatory and anticancer effects through modes of crosstalk between NF-κB and other signaling molecules

### 3.1 Mechanism of MCL in NF-κB signaling pathway and other pathways

MCL has been synthesized to overcome the instability of PTL under acidic and basic conditions. MCL is a natural guaianolide sesquiterpene lactone found in Michelia compressa and Michelia champaca, and can also be synthesized from PTL *in vitro*. MCL exhibits anti-inflammatory and anticancer activities similar to PTL but is more stable under both *in vitro* and *in vivo* conditions (Peng et al., 2019). MCL inhibits NF-κB activation by alkylating p65 cysteine-38, preventing DNA binding and exerting an antagonist effect on NF-κB (Ghantous et al., 2013). Given NF-κB’s pivotal role in inflammation and immune responses, MCL’s ability to modulate this pathway holds significant therapeutic potential in treating inflammatory diseases such as RA and inflammatory bowel disease (Viennois et al., 2014).

Besides, MCL exerts anti-inflammatory effects by modulating the PI3K/Akt signaling pathway. In Alzheimer’s disease (AD) model mice, MCL improves cognitive function and reduces brain inflammation by inhibiting glial cell activation and cytokine secretion. These effects are linked to the reduction of Akt phosphorylation and the downregulation of key inflammatory mediators (Yang G. et al., 2023). Similarly, in a doxorubicin (DOX)-induced cardiotoxicity model, MCL mitigates cardiac injury, improves cardiac function, and alleviates histopathological damage through the regulation of the PI3K/Akt pathway, decreasing phosphorylated Akt, Bad, and caspase-3 levels, while increasing phosphorylated PTEN (Kalantary-Charvadeh et al., 2019). In addition to its effects on the NF-κB signaling pathway and the PI3K/Akt pathway, MCL also activates the MAPK signaling pathway in cancer and fibrotic models. In pancreatic and colon cancer cells, MCL enhances ROS production and regulates cell death mechanisms such as autophagy, paraptosis, and ferroptosis via MAPK activation (Yang M. H. et al., 2024). Furthermore, MCL suppress the Mtdh/BMP/MAPK pathway in renal fibrosis, reducing fibrotic markers and restoring epithelial function in both *in vivo* and *in vitro* models (Peng et al., 2019).

### 3.2 Anti-inflammatory effects of MCL in disease models

Inflammatory factors are pivotal in inflammatory diseases progression and significantly influence the efficacy of therapies (Zhai et al., 2024; Xiao et al., 2019; Xiao et al., 2020; Zhang et al., 2024; Ni et al., 2021; Zhang et al., 2023a; Ni et al., 2020). The NF-κB signaling pathway has been directly implicated in the development of various inflammation-associated disorders, as demonstrated in studies using transgenic mouse models. In a dextran sodium sulfate (DSS)-induced murine model of colitis, DSS treatment resulted in a significant increase in the expression levels of pro-inflammatory cytokines such as IL-1β, TNF-α, and IL-6, which play key roles in the pathogenesis of inflammatory bowel disease (IBD). Treatment with MCL was shown to significantly reduce the DSS-induced release of these cytokines by modulating the NF-κB signaling pathway (Viennois et al., 2014). Similarly, these cytokines are central to the pathophysiology of rheumatoid arthritis (RA) (Chen et al., 2019). The therapeutic effects of MCL in RA were further confirmed in a murine model, with potential mechanisms including alterations in the expression of cytokines such as C5/C5a, TIMP-1, M-CSF, and BLC (Xu et al., 2015).

Sepsis, another inflammation-associated condition, is triggered when lipopolysaccharide binds to TLR4, initiating the production of large quantities of inflammatory cytokines, which play a pivotal role in the onset of sepsis (Savva and Roger, 2013). In addition to its effect on NF-κB activation, MCL has been shown to reduce the levels of various LPS-induced inflammatory cytokines by inhibiting the phosphorylation of p70S6K (Thr389) and Akt (Ser473) (Qin et al., 2016). Moreover, a recent study highlighted MCL’s therapeutic potential in sepsis caused by Gram-positive bacteria and antibiotic-resistant bacteria through similar mechanisms (Jiang et al., 2017). Further investigation into MCL’s regulatory effects on the immunopathology of tuberculosis, induced by *Mycobacterium tuberculosis* (Mtb), reinforced its therapeutic potential in treating inflammation-related disorders. MCL dose-dependently reduced the secretion of inflammatory cytokines such as TNF-α and IL-1β in response to Mtb. Additionally, MCL inhibited Mtb-induced phosphorylation of Akt (Ser473) in Raw264.7 cells, thereby attenuating the activation of the NLRP3 inflammasome (Zhang et al., 2017).

Recent studies have also expanded on the therapeutic potential of MCL in other inflammation-related diseases. A study synthesizing novel ferulic acid-michelin lactone (FA-MCL) derivatives found that one such derivative exhibited significant anti-inflammatory activity in LPS-induced inflammatory responses, suggesting its potential as a candidate drug for acute lung injury (ALI) (Duan et al., 2024). Additionally, MCL has been shown to alleviate severe acute pancreatitis (SAP)-induced pancreatic injury by activating Nrf2-regulated antioxidant pathways and inhibiting NF-κB p65-mediated inflammation, further emphasizing its anti-inflammatory therapeutic potential in SAP (Wu et al., 2024). As a neuroprotective agent, MCL may also inhibit neuroinflammation in neurodegenerative disorders (Sun et al., 2017). In Alzheimer’s disease (AD), MCL mitigates neuroinflammation by inhibiting the NF-κB and PI3K/Akt pathways in glial cells, leading to a reduction in inflammatory mediators and improvements in cognitive function, positioning MCL as a promising therapeutic candidate for AD (Yang G. et al., 2023). Furthermore, by promoting autophagy and facilitating the degradation of the NLR pyrin domain three (NLRP3) inflammasome, MCL reduces inflammation and tissue damage, offering potential as a novel anti-inflammatory therapy for radiation-induced enteropathy (RIE) (Wu et al., 2021).

### 3.3 Anticancer activities of MCL across different cancer types

Immune microenvironment is pivotal in tumor progression (Yang H. et al., 2024; Sun Z. et al., 2024; Wang J. et al., 2023), and significantly influence the efficacy of cancer therapies (Deng et al., 2024; Xia et al., 2023; Sun L. et al., 2024; Zhang J. et al., 2023; Li X. et al., 2024). The NF-κB signaling pathway, a central component of the inflammation-fibrosis-cancer axis, is implicated not only in inflammatory diseases but also in pathological states where inflammation serves as a fundamental factor, such as in various forms of cancer (Czauderna et al., 2019; Zhao et al., 2023). The transition from inflammation to cancer may be mediated by several regulatory molecules (Wang Y. et al., 2023; Wang et al., 2025), including chemokines, proinflammatory cytokines, TNF, and IL-6, which are crucial in promoting the growth, proliferation, and invasion of cancer cells (Singh et al., 2019; Jiang et al., 2024; Zhang et al., 2023c; Zhao et al., 2024; Shao et al., 2024; Wang et al., 2024). Notably, colitis-associated cancer (CAC), which arises from chronic colonic inflammation, highlights the potential therapeutic role of MCL in this context (Viennois et al., 2014). Specifically, IKKβ-induced NF-κB activation in enterocytes plays a significant role in tumor initiation during the early stages, while IKK2-mediated NF-κB activity in myeloid cells promotes tumor progression by stimulating the expression of proinflammatory cytokines that act as tumor growth factors (Greten et al., 2004). Therefore, MCL could be considered a potential therapeutic agent for treating CAC through its inhibitory effects on NF-κB.

Hepatocellular carcinoma (HCC) is another inflammation-driven cancer (Zhai et al., 2023; Zhang S. et al., 2023; Zhang et al., 2023e), where inflammatory cytokines such as TNF-α and IL-6 activate downstream targets including NF-κB, JNK, and STAT3, thereby promoting tumorigenesis (Czauderna et al., 2019; Yang et al., 2019; Kumari et al., 2016; Kitamura et al., 2017). MCL, a natural thioredoxin reductase (TrxR) inhibitor, has emerged as a promising therapeutic agent for HCC. It induces immunogenic cell death (ICD) in HCC cells via the generation of reactive oxygen species (ROS) and the induction of endoplasmic reticulum (ER) stress (Xu et al., 2022) (Table 2). The anticancer efficacy of MCL is primarily attributed to its specific inhibition of TrxR, which results in the accumulation of ROS and the promotion of oxidative stress-induced apoptosis in malignant cells. MCL covalently binds to the Sec residue at position 498 of TrxR, thereby impairing its biological function, reducing TrxR activity, increasing oxidized Trx, and accumulating ROS. This disruption of redox homeostasis sensitizes cancer cells to ionizing radiation (IR) and enhances their cytotoxic response. Mechanistic studies have further suggested that MCL’s inhibition of TrxR contributes significantly to enhanced radiosensitivity, offering potential as both a chemotherapeutic agent and a radiosensitizer in cancer treatment (Zhang et al., 2022). In a xenograft liver cancer model, MCL was found to inhibit tumor growth by promoting apoptosis and disrupting the actin cytoskeleton (Yu et al., 2019). Thus, MCL presents substantial therapeutic potential for the treatment of liver cancer.

**TABLE 2:** Anti-tumor mechanisms involving induction of cell death and apoptosis.

Compound	Cell death mechanism	Effect	Cancer type	Ref
PTL	Apoptosis induction	Induces apoptosis in various cancer cells	Acute Lymphoblastic Leukemia, Melanoma, Lung Cancer, Colorectal Cancer, Pancreatic Cancer	Ortiz-Reyes et al. (2024), Dorostgou et al. (2024), Cai et al. (2024), Liu et al. (2017a), Liu et al. (2017b), Denda et al. (2024)
PTL	Migration and invasion effects	Inhibits cancer cell migration and invasion	Colorectal Cancer	Zhu et al. (2019), Kim et al. (2017a)
MCL	Autophagy, Paraptosis, Ferroptosis	Induces autophagy, paraptosis, ferroptosis through MAPK signaling and ROS production	Hepatocellular Carcinoma	Yang et al. (2024a), Xu et al. (2022)
MCL	Reactive oxygen species (ROS) and endoplasmic reticulum (ER) stress induction	Increases ROS levels and ER stress	Hepatocellular Carcinoma	Xu et al. (2022)
MCL	Glutathione (GSH) Depletion	Depletes GSH, disrupts mitochondrial function, enhances cancer cell death	Leukemia, Glioblastoma	Guo et al. (2022)
DMAMCL	Apoptosis via Bcl-2 Downregulation	Lowers Bcl-2, elevates apoptosis in glioma cells	Glioma	An et al. (2015)
DMAMCL	Autophagy, Paraptosis, Ferroptosis	Induces autophagy, paraptosis, ferroptosis through MAPK signaling and ROS production	Pancreatic Cancer, Colon Cancer	Deng et al. (2024)
DMAMCL	Glutathione (GSH) Depletion	Depletes GSH, disrupts mitochondrial function, enhances cancer cell death	Leukemia, Glioblastoma	Jiang et al. (2024)
DMAMCL	Apoptosis and Autophagy	Induces apoptosis and autophagy by regulating ROS/MAPK and Akt/mTOR pathways	U87-MG and U251 Cell Lines	Wang et al. (2019a)
DMAMCL	Differentiation Induction	Promotes differentiation, inhibits growth in rhabdomyosarcoma and osteosarcoma stem cells	Rhabdomyosarcoma, Osteosarcoma	Li et al. (2024b), Ba et al. (2020), Xu et al. (2019)

Gastric carcinoma, originating from the epithelium of the gastric mucosa, is one of the most prevalent malignancies globally, with an increasing incidence, particularly among younger populations, largely due to dietary changes, rising work-related stress, and *Helicobacter pylori* infection. The primary treatment for advanced gastric cancer involves a combination of neoadjuvant chemoradiotherapy, molecular-targeted therapy, and immunotherapy (Song et al., 2017). However, prognosis remains poor for patients with advanced disease (Tirino et al., 2018), highlighting the critical need to improve therapeutic outcomes. Recent studies have indicated that IL-6 is upregulated in gastric cancer tissues, serving as a marker for poor prognosis. This upregulation is associated with IL-6’s role in promoting tumor cell invasion, metastasis, and angiogenesis (Unver and McAllister, 2018). Additionally, a mouse study on gastric tumorigenesis induced by N-methyl-N-nitrosourea revealed that IL-6 promotes gastric cancer cell proliferation through the activation of STAT3, as demonstrated by comparing IL-6 knockout mice with wild-type mice (Kinoshita et al., 2013). Targeting and blocking the IL-6/STAT3 pathway therefore represents an effective therapeutic strategy for gastric cancer. Studies on MCL have shown that it suppresses gastric cancer growth by blocking the IL-6/STAT3 pathway (Tang et al., 2019). Specifically, in both cellular and *in vivo* studies, treatment with MCL resulted in a reduction of IL-6 expression and inhibited the STAT3 signaling pathway. Furthermore, the restoration of the STAT3 pathway through the addition of exogenous IL-6 supports the notion that MCL exerts its anti-cancer effects by downregulating IL-6, thereby blocking STAT3 activation (Tang et al., 2019). Hence, MCL not only holds potential as a treatment for gastric cancer but could also improve the prognosis of patients with advanced gastric cancer.

Beyond its impact on previously discussed malignancies, MCL exhibits considerable therapeutic promise across a range of other cancers, including pancreatic, hematologic, and breast cancers. In pancreatic and colon cancer cell lines, MCL elicits anticancer responses by stimulating autophagy, paraptosis, and ferroptosis, processes that are mediated through the activation of MAPK signaling and the generation of reactive oxygen species (ROS) (Xu et al., 2022). Moreover, MCL augments the effectiveness of ruxolitinib in managing myeloproliferative neoplasms (MPNs) by targeting the JAK/STAT signaling cascade. This enhancement is accomplished through covalent attachment to cysteine residues on STAT3 and STAT5, leading to diminished phosphorylation and a reduction in mutant allele burden in Jak2V617F-mutated models (Huang et al., 2023). The active metabolite of MCL, known as ACT001, demonstrates antitumor activity in pancreatic ductal adenocarcinoma (PDAC) by restraining cell proliferation, promoting apoptosis, and increasing ROS levels. The fundamental mechanism encompasses modulation of the EGFR-Akt-Bim signaling pathway, as observed in both *in vitro* and *in vivo* studies (Yang J. et al., 2023). In leukemia and glioblastoma cell types, MCL elicits strong anticancer effects by initiating oxidative stress, mainly via the depletion of glutathione (GSH), thereby impairing mitochondrial function and promoting cancer cell apoptosis. The combination with GSH biosynthesis inhibitors, such as L-buthionine sulfoximine (BSO), intensifies this effect, presenting a viable approach to surmount resistance to conventional cancer treatments (Guo et al., 2022). Additionally, MCL has been demonstrated to counteract tamoxifen resistance in breast cancer (BC) by downregulating the expression of Amidohydrolase 1 (ASAH1), adjusting the ROS/AKT signaling pathway, and activating the NRF2/KEAP1 antioxidant mechanism. This results in the suppression of cell proliferation and improved therapeutic effectiveness against tamoxifen-resistant BC cells (Han et al., 2024). In summary, MCL exhibits extensive anticancer potential through the activation of multiple cell death pathways, the augmentation of other therapeutic modalities, and the ability to overcome drug resistance, thereby establishing it as a multifaceted candidate for cancer therapy.

## 4 DMAMCL: an anti-inflammatory and anticancer prodrug derived from MCL

### 4.1 Pharmacokinetics and stability of DMAMCL

DMAMCL, the dimethyl amino Michael adduct of MCL, demonstrates superior stability, enhanced activity, and reduced toxicity compared to MCL. When administered orally, DMAMCL exhibits a significantly longer half-life than its counterpart, DMAPT, and exerts potent anti-inflammatory effects (Viennois et al., 2014). As previously discussed, MCL has shown efficacy in treating inflammatory bowel diseases and other inflammatory conditions. Furthermore, DMAMCL has also been identified as a promising therapeutic agent for diabetic kidney disease (DKD) and neuroinflammation, exhibiting effects similar to those of MCL. Notably, DMAMCL has been observed to induce downregulation of metadherin (Mtdh), thereby inhibiting NF-κB signaling and suppressing downstream inflammatory cytokines. This mechanism suggests that DMAMCL could attenuate Mtdh-mediated renal inflammation in DKD (Liu et al., 2019). Additionally, DMAMCL has been reported to alleviate NLRP3-mediated neuroinflammation, potentially inhibiting the progression of Parkinson’s disease (PD) by modulating the NLRP3 inflammasome (Liu et al., 2020b).

### 4.2 Therapeutic efficacy of DMAMCL in cancer treatment

#### 4.2.1 Hepatocellular carcinoma (HCC)

DMAMCL metabolizes slowly in plasma, gradually releasing MCL as its active metabolite, which contributes to its improved pharmacokinetic properties and potential as a promising anticancer agent. In hepatocellular carcinoma (HCC), DMAMCL triggers apoptosis and induces cell cycle arrest at the G2/M phase by activating reactive oxygen species (ROS) production and inhibiting the PI3K/Akt pathway (Yao et al., 2020). Thus, DMAMCL presents as a viable therapeutic candidate for the treatment of HCC.

#### 4.2.2 Glioblastoma (GBM)

Glioblastoma (GBM), recognized as one of the most malignant and aggressive primary brain tumors, presents significant treatment challenges. Standard therapy, which includes surgical resection followed by radiotherapy and chemotherapy with temozolomide (TMZ), often results in poor prognosis due to tumor heterogeneity, limited central nervous system (CNS) penetration, and drug resistance (Zanders et al., 2019). Consequently, there is an urgent need for new therapeutic agents or combination therapies to address these obstacles. DMAMCL is currently undergoing phase I clinical trials for the treatment of GBM (trial ID: ACTRN12616000228482) and has been designated as an orphan drug for GBM by the FDA. Previous studies have elucidated the mechanisms by which MCL targets gliomas, particularly its interaction with PAI-1, a direct target of DMAMCL. ACT001, a metabolite of DMAMCL, binds to PAI-1, thereby inhibiting the PAI-1/PI3K/AKT pathway, which in turn suppresses glioma cell proliferation, invasion, and migration. Additionally, DMAMCL has been shown to inhibit STAT3 phosphorylation by directly binding to STAT3, reducing PD-L1 expression in glioma cells, as phosphorylated STAT3 binds to the PD-L1 promoter to modulate its transcription (Zanders et al., 2019; Wang et al., 2020). Furthermore, MCL, released from DMAMCL, selectively targets monomeric PKM2 and promotes its tetramerization, thereby altering the glycolytic metabolism of GBM cells and suppressing their proliferation (Guo et al., 2019). Additionally, DMAMCL may target the Bcl-2 signaling pathway, further enhancing its antitumor efficacy in glioma cells (An et al., 2015). Finally, DMAMCL induces apoptosis and autophagy through regulation of the ROS/MAPK and Akt/mTOR signaling pathways in U87-MG and U251 glioblastoma cell lines (Wang Y. et al., 2019).

#### 4.2.3 Rhabdomyosarcoma (RMS) and osteosarcoma

DMAMCL promotes differentiation and inhibits the growth of rhabdomyosarcoma (RMS) by inducing morphological changes, enhancing muscle differentiation markers, and downregulating DLL1, a key ligand in the Notch signaling pathway. This positions DMAMCL as a potential agent for differentiation-based therapies in RMS (Li Q. et al., 2024). In addition, DMAMCL has demonstrated promising antitumor effects in RMS and osteosarcoma, the most common soft tissue sarcoma and primary bone malignancy, respectively, particularly in pediatric and adolescent populations. The underlying mechanism of DMAMCL’s efficacy in these cancers is likely associated with its induction of Bim, modulation of the NF-κB pathway, ROS generation in RMS cell lines, and reduction of osteosarcoma stem cell stemness (Ba et al., 2020; Xu et al., 2019).

#### 4.2.4 Acute myeloid leukemia (AML)

In acute myeloid leukemia (AML), DMAMCL has been shown to decrease the percentage of stem cells in primary AML cells (Zhang et al., 2012). As mentioned earlier, MCL targets PKM2 and induces its irreversible tetramerization, which suppresses the proliferation of GBM cells. Furthermore, DMAMCL significantly inhibits leukemia cell growth as a PKM2-targeted therapeutic agent (Li J. et al., 2018).

In conclusion, DMAMCL represents an eco-friendly, less toxic alternative to many conventional chemical anticancer agents. Given its promising therapeutic profiles across various cancers, DMAMCL could emerge as a versatile, effective compound for cancer treatment.

## 5 Advantages of Dimethylaminomicheliolide (DMAMCL)

DMAMCL has been extensively characterized and compared with other natural compounds, such as MCL and PLT. The published findings indicate that DMAMCL offers several advantages over both MCL and PLT. Notably, DMAMCL has been identified as a potent suppressor of GBM cell proliferation by targeting pyruvate kinase M2 (PKM2) and facilitating the rewiring of aerobic glycolysis (Guo et al., 2019). In the context of peritoneal fibrosis (PF), a primary cause of ultrafiltration failure in patients undergoing long-term peritoneal dialysis, DMAMCL emerges as a promising new compound due to its low toxicity, high stability, and sustained release of MCL, presenting a significant advantage in terms of safety and efficacy (Li et al., 2019). Furthermore, research has demonstrated that the LC50 values of DMAMCL against C6 and U-87MG glioma cell lines *in vitro* were 27.18 ± 1.89 μM and 20.58 ± 1.61 μM, respectively. Importantly, DMAMCL was found to significantly reduce the expression of the anti-apoptotic gene Bcl-2 and promote apoptosis in C6 and U-87MG cells in a dose-dependent manner. These findings suggest that DMAMCL holds considerable potential for the treatment of glioma (An et al., 2015).

## 6 Combination therapy for various cancers involving PTL, MCL, and DMAMCL

Combination therapy has been shown to provide superior therapeutic effects compared to monotherapy by inhibiting tumor growth and improving survival rates (Zhou et al., 2021; Liu et al., 2022; Li et al., 2023; Zhang et al., 2023f). Moreover, long-term high-dose monotherapy often results in severe side effects and the development of drug resistance (Jin et al., 2024). Consequently, combining two or more drugs can enhance therapeutic efficacy, reduce adverse drug reactions, and target various symptoms or comorbidities. In recent years, PTL, MCL, and DMAMCL have demonstrated significant potential in combination therapy, with promising applications in the treatment of various diseases and in improving existing treatment regimens.

The mechanisms and therapeutic effects of PTL in treating various diseases have been extensively studied. Notably, the synergistic benefits of PTL in combination with other drugs have gradually been recognized as more effective than monotherapy. In human acute myeloid leukemia (AML) cells, combining PTL with pan-histone deacetylase inhibitors (HDACIs) potentiates HDACI lethality by inhibiting NF-κB and the subsequent activation of the SAPK/JNK pathway via MKK7 (Dai et al., 2010). Moreover, it was found that PTL treatment upregulates NADPH levels via the pentose phosphate pathway (PPP) and activates Nrf2-mediated responses. To overcome these challenges, a novel triple-drug regimen known as PDT—comprising 2-deoxyglucose, PTL, and temsirolimus—has been designed and shown to exhibit potent therapeutic effects on primary AML cells, including those resistant to PTL alone (Pei et al., 2016).

Balsalazide, serving as a prodrug for 5-aminosalicylate, has exhibited promise in chemoprevention for patients with ulcerative colitis within the scope of treating human colorectal cancer and colitis-associated cancer (CAC) (Do et al., 2016). The simultaneous use of PTL with balsalazide leads to a synergistic inhibition of NF-κB activation, thereby enhancing therapeutic efficacy (Kim et al., 2017b). In the MDA-MB-468 breast cancer cell line, epirubicin promotes apoptosis and demonstrates anticancer properties, yet it also induces cytotoxic effects in normal cells. Combining PTL with epirubicin achieves similar antiproliferative and proapoptotic outcomes while reducing cytotoxicity in healthy cells, thereby mitigating side effects (Ghorbani-Abdi-Saedabad et al., 2020). Triple-negative breast cancer (TNBC) presents the poorest prognosis and the highest risk of distant metastasis among all breast cancer subtypes, highlighting the need for innovative strategies to improve tumor sensitivity to treatments. Research by Carlisi et al. showed that the combination of SAHA and PTL increased the sensitivity of MDA-MB-231 breast cancer cells to PTL’s cytotoxic effects (Carlisi et al., 2015). Moreover, PTL was demonstrated to prevent TNBC cells from developing resistance to doxorubicin and mitoxantrone by suppressing the overexpression of Nrf2 (Carlisi et al., 2017).

Recent research also highlighted the potential of combination therapy in treating advanced hepatocellular carcinoma (HCC). The PTL nanocrystal delivery system, in combination with sorafenib (Sora), was used to treat tumors in mice with HCC, achieving a tumor inhibition rate of 81.86%, significantly higher than that of either PTL or Sora alone, indicating the combination’s ability to enhance the therapeutic effect while overcoming the poor water solubility of PTL (Liang et al., 2020). Moreover, PTL has been reported to enhance the sensitivity of gastric cancer cells to cisplatin (DDP) and to reverse drug resistance by inhibiting the STAT3 signaling pathway (Li H. et al., 2018). In the treatment of glioblastoma multiforme (GBM), temozolomide (TMZ) remains the standard chemotherapeutic drug, but its effectiveness is limited by chemoresistance. Inhibition of NF-κB has been shown to enhance anti-glioma activity and reduce TMZ-induced resistance by downregulating MGMT gene expression (Yu et al., 2018).

Given that DMAMCL, MCL, and PTL alone may not fully eradicate GBM, combination treatments involving ACT001 with other therapies have been explored. ACT001, in combination with cisplatin, has shown synergistic effects by suppressing the PAI-1/PI3K/AKT pathway, enhancing apoptosis in glioma cells and overcoming cisplatin resistance (Xi X. et al., 2019). DMAMCL is capable of crossing the blood-brain barrier (BBB) and accumulating in the brain (Xi X. N. et al., 2019), leading to decreased PD-L1 expression. Although side effects need to be addressed, DMAMCL, when combined with TMZ, may serve as an optimal treatment for GBM, reversing immunosuppression and inhibiting immune escape in GBM cells induced by TMZ (Wang S. et al., 2019). In Parkinson’s disease (PD), L-3,4-dihydroxyphenylalanine (L-DOPA) is the primary treatment, though long-term use may result in severe side effects. Studies have shown that DMAMCL, when administered in conjunction with a low dose of L-DOPA, yields therapeutic effects equivalent to those of high-dose L-DOPA in mouse models of PD, suggesting that DMAMCL enhances the curative effect of L-DOPA (Liu et al., 2020c). Furthermore, the combination of MCL and radiotherapy has been shown to improve the sensitivity of cancer tissues to radiation, enhancing the therapeutic effect. MCL promotes radiosensitivity in p53-deficient non-small cell lung cancer (NSCLC) by inhibiting the HIF-1α pathway, thereby promoting its degradation (Kong et al., 2020). Additionally, MCL-mediated depletion of glutathione (GSH) reduces KLF4-mediated cisplatin resistance in breast cancer cells (Jia et al., 2015).

## 7 Conclusion

Inflammation plays a significant role in tumor initiation and progression, with carcinogens in colon cancer and inflammatory bowel disease serving as prominent examples. Over the years, various biological macromolecules have been identified and developed as anticancer and anti-inflammatory agents to address these issues and associated infections. Notably, PLTs have achieved extensive application in the treatment of cancer and inflammatory disorders via modulating key signaling pathways, including NF-κB, STAT3, and MAPK, which are essential to both oncogenic progression and the regulation of immune responses. This review underscores the ability of PLTs to shield normal cells from apoptosis while selectively triggering apoptotic mechanisms in cancerous cells. Consequently, the findings presented herein offer significant insights that may drive future investigations, potentially leading to the development of targeted therapies that leverage the anticancer and anti-inflammatory properties of PLTs.
